# Tunneling-injection in vertical quasi-2D heterojunctions enabled efficient and adjustable optoelectronic conversion

**DOI:** 10.1038/srep31475

**Published:** 2016-08-10

**Authors:** Wei-Chun Tan, Chia-Wei Chiang, Mario Hofmann, Yang-Fang Chen

**Affiliations:** 1 Department of Physics, National Taiwan University, Taipei, 10617 Taiwan; 2 Department of Material Science and Engineering, National Cheng Kung University, Tainan, 70101 Taiwan

## Abstract

The advent of 2D materials integration has enabled novel heterojunctions where carrier transport proceeds thrsough different ultrathin layers. We here demonstrate the potential of such heterojunctions on a graphene/dielectric/semiconductor vertical stack that combines several enabling features for optoelectronic devices. Efficient and stable light emission was achieved through carrier tunneling from the graphene injector into prominent states of a luminescent material. Graphene’s unique properties enable fine control of the band alignment in the heterojunction. This advantage was used to produce vertical tunneling-injection light-emitting transistors (VtiLET) where gating allows adjustment of the light emission intensity independent of applied bias. This device was shown to simultaneously act as a light detecting transistor with a linear and gate tunable sensitivity. The presented development of an electronically controllable multifunctional light emitter, light detector and transistor open up a new route for future optoelectronics.

The discovery of graphene and other 2D materials has led to the emergence of a new class of hybrid materials that derive their properties from interfaces between layers of dissimilar materials^1^,^2^,^3^,^4^. Carrier transport in such heterojunctions is commonly proceeds by tunneling and thermionic emission current which is fundamentally different from traditional diffusion-based charge transport and makes these structures especially promising for optoelectronic devices^5^,^6^,^7^,^8^,^9^,^10^,^11^. Conversion between electrical and optical signals is required in a large variety of applications ranging from solid state lighting, ubiquitous information displays, light-based wireless communication and optical computing^12^,^13^,^14^,^15^,^16^. Future development in these areas relies on improvements in several areas.

A wide range of accessible wavelengths, for example, is required for lighting applications as well as wide-bandwidth optical communication. Furthermore, the increasing resolution of displays will necessitate high integration densities of emitters^17^,^18^,^19^,^20^. Finally, optical computing applications will require multifunctional devices that allow efficient coupling between electronic and optical functionalities. All of these advances will have to be compatible with increased scale and ease of fabrication.

Current light emitters have severe limitations that prevent them from supporting these developments. While crystalline semiconductors exhibit composition-dependent emission properties, fabrication challenges arise for each new material combination^21^. Furthermore, emission characteristics are deteriorated by large bias currents and inevitable defects^22^. While organic semiconductors promise low cost production and access to different emission profiles their intrinsically slow carrier transport limits the efficiency of those emitters^23^,^24^,^25^. Finally, future requirements for higher device integration density, e.g. in monolithically integrated light emitting diode (LED) arrays and on-chip optical communication, cannot be satisfied with current configurations where each light emitter is driven by a dedicated transistor^26^.

We here demonstrate the potential of metal/insulator/semiconductor heterojunctions between 2D and thin film materials as a composited architecture for efficient and multifunctional optoelectronic devices as shown in Fig. 1(a). The tunneling-based carrier transport produces efficient and color stable light emission. The operation mechanism was elucidated to be a gate-tunable and energy-selective carrier tunneling process, which is different from previously reported graphene-based Schottky-barrier devices^27^ and represents an operation mechanism that is compatible with many different luminescent materials. In the case of GaN selective injection in states close to the band edge results in a narrow emission peak at 400 nm. The demonstrated electrostatic control over the emission intensity through gating yields three-terminal vertical tunneling-injection light emitting transistors (VtiLET) with high efficiency. Our architecture furthermore enables the application of such light emitters as a photodetector with linear and tunable sensitivity. Finally, the employed self-aligned vertical architecture significantly reduces fabrication complexity and is suitable for a wide range of morphologies and nanostructures.

## Results

### The electroluminescence and I-V characteristics of VtiLET device

**Figure 1:**
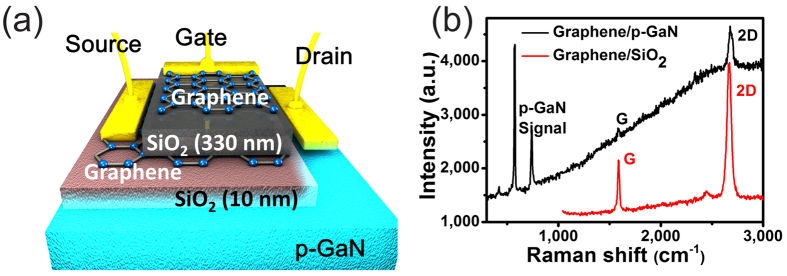
(**a**) Schematic of the vertical tunneling injection light emitting transistor (VtiLET) (**b**) Micro-Raman spectra for graphene on p-GaN and SiO_**2**_ substrates with the indication of p-GaN and graphene characteristic peaks.

**Figure 2:**
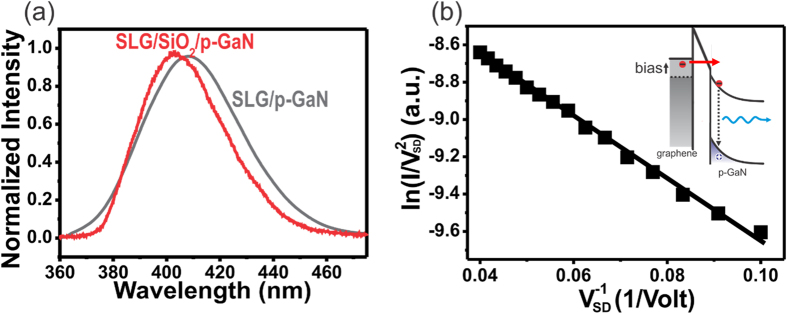
(**a**) Electroluminescence (EL) spectra of graphene/thin SiO_2_/p-GaN device under forward bias (V_SD_ > 0 V) of 12 V. (**b**) Simmons plot of current measurement and the measured data were fitted to equation (1). (inset) Energy level diagram of the graphene/thin SiO_2_/p-GaN device under forward bias (V_SD_ > 0 V).

The fabricated vertical tunneling-injection light emitting transistor on sapphire substrate with the structure shown in Fig. 1(a) was characterized using Raman spectra to reveal the signals of p-GaN and graphene separately in Fig. 1(b). The top layer of graphene plays a role of gate electrode. Meanwhile, the middle layer of graphene is the source terminal and the drain terminal belongs to p-GaN. In order to demonstrate the importance of the thin 10 nm SiO_2_ layer in the emission process, we first compare the result for both samples of graphene/SiO_2_/p-GaN and graphene/p-GaN. Figure 2(a) shows the electroluminescence (EL) spectra of the graphene/SiO_2_/p-GaN and graphene/p-GaN devices under forward source-drain-bias (which was defined V_D_ > 0 V, V_S_ = 0 V, and then V_SD_ > 0 V here). The emission peak of graphene/SiO_2_/p-GaN device around 400 nm can be attributed to the transition from states near the conduction band to acceptor impurity states caused by interstitial Mg atoms near the valence band^28^. A small shoulder at around 387 nm originates from p-GaN band edge emission around 3.2 eV^29^. The emission behavior is different from commonly observed emission in metal/insulator/semiconductor devices that operate through impact ionization and avalanche multiplication and require large reverse-bias voltages^30^,^31^,^32^. Furthermore, the emission spectrum in graphene/p-GaN heterojunctions (without thin SiO_2_) is characterized by a broad band emission as shown in Fig. 2(a).

The difference in emission characteristics can be explained when considering the added oxide layer. Without the oxide layer, the applied voltage will mainly drop in the GaN film, which can induce a large band bending near the Schottky interface and cause a broad emission and red shift. However, with the addition of the oxide layer, the highest potential drop will occur at the tunneling interface and minimal band bending within the p-GaN. Under an applied forward bias (V_SD_ > 0 V), the bands of p-GaN at the interface near the thin SiO_2_ are bent upwards and the work function of graphene changes to smaller values (inset of Figure 2(b)). Due to the large voltage drop across the thin SiO_2_ insulating layer, the applied bias induces an accumulation layer of holes near the SiO_2_/p-GaN interface. The injected electrons can then recombine radiatively with holes in p-GaN near the interface and generate the UV emission, which will reduce the linewidth and cause the blue shift compared with the sample without thin SiO_2_ layer.

In the tunneling regime, the Simmon’s approximation describes the current-voltage diagram. In the applicable limit of large applied bias, the dependence equation of I_SD_ versus V_SD_ can be written as^33^,^34^,


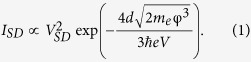


Here d is the barrier width, m_e_ is the electron effective mass and φ is the tunneling barrier height. The good agreement with the model is demonstrated in Fig. 2(b), where we plot the natural logarithm of the source-drain current divided by 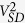 as a function of 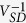. As expected, the plot exhibits a linear dependence which is commonly known as Fowler-Nordheim tunneling. As shown in Fig. 2(b), the field emission goes through a triangular barrier under a large voltage^35^.

The observed mechanism is fundamentally different from the traditional operation of an LED. While conventional LEDs operate through minority carrier conduction and recombination, our tunneling-LED works by injecting carriers directly into prominent states by tunneling without the need for efficient carrier transport. Therefore tunneling-LEDs are compatible with a wide range of luminescent materials that do not exhibit high carrier conductivities. Furthermore, the large potential drop across the oxide barrier minimizes the band bending within the p-GaN which reduces the effect of impact ionization that usually broadens the spectrum. Consequently, a 13% narrowing full width at half maximum (FWHM) and 5.4 nm blue shift of the p-GaN emission spectrum can be observed (Fig. 2(a)).

### The electric field effect performance of VtiLET device

**Figure 3:**
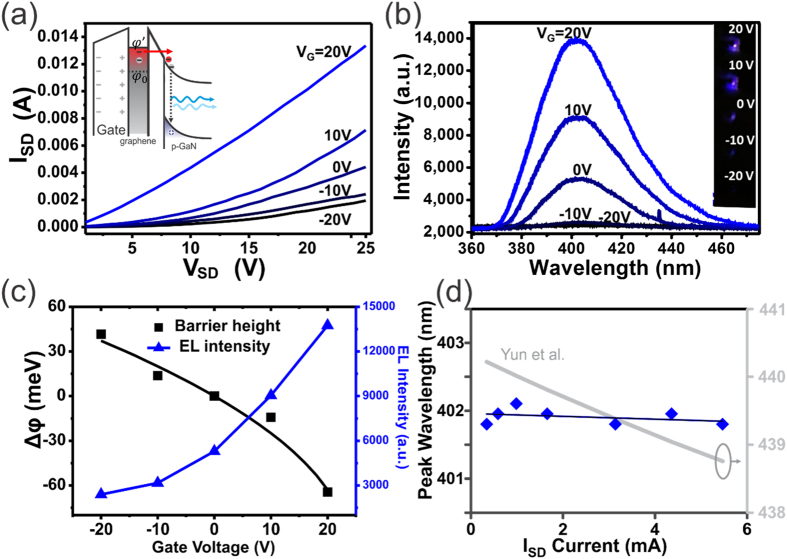
(**a**) The characteristic of I_SD_ versus V_SD_ at varying gate voltages V_G_. (inset) Energy level diagram of the graphene/thin SiO_2_/p-GaN device under a positive external gating (V_G_) and a forward bias (V_SD_ > 0 V). The work function of graphene will decrease from φ_0_ to *φ*′ der positive external gate voltage, (**b**) Electroluminescence (EL) spectra of graphene/thin SiO_2_/p-GaN device under a fixed forward bias (V_SD_ = 12 V) at different gate voltages. The inset shows a series of pictures of the light emission evolution under respective gate voltage (V_G_). (**c**) Extracted barrier height and EL intensity as a function of gate voltage (V_G_), (**d**) emission peak wavelength at different emission currents with comparison of previously reported GaN emission wavelength^22^.

The single layer graphene serves in a two-fold role in our architecture. First, its very small effective electron mass improves the efficiency of tunneling injection^2^,^36^. Furthermore, graphene acts as a transparent electrode that allows 3x more efficient emission of the UV light compared to common ITO electrodes (see Figure S1 for more details). Another feature of graphene is subsequently exploited to enhance the functionality of the emitter. Graphene’s work function can be widely tune through application of an electrostatic gate (V_G_)^37^. We observe that the tunneling current shows a clear dependence on applied electrostatic gating (V_G_) (Fig. 3(a)). To compare the I_SD_ with bias voltage V_SD_ at 5 V from these I_SD_-V_SD_ curves, the device performance as a transistor under the gate voltage (V_G_) is changed from −20 V to 20 V. We observe an on/off ratio of 10^2^ indicating effective suppression of current flow even at large forward biases. Previous reports have shown the high achievable switching speed for graphene-based tunneling transistors which indicates the potential of our approach for high speed emitters^7^.

We now turn to identifying the effect of electrostatic gate control on the light emission process. Figure 3(b) shows the EL spectra and a series of photos of the device under different gating voltages (V_G_). Under positive gate voltage, the device shows higher light emission than that at zero gate voltage. On the other hand, suppressed emission can be observed under negative gate voltage (V_G_). This behavior shows that our emitter can be operated in a three-terminal configuration as a light emitting transistor. This ability could be exploited in compact light emitters through integration in crossbar matrix arrays for high resolution displays.

The demonstrated light emitting transistor is different from previous LET structures where a gate voltage was used to modulate the carrier transport along a semiconducting channel^26^. The difference is apparent when analyzing the operation mechanism. We extracted the barrier height of the tunneling emission as a function of gate voltage from the Simmon’s-approximation. A clear decrease in barrier height with applied gate is shown in Fig. 3(c). This behavior suggests that graphene under positive gate voltage would have a smaller work function than before and thus cause a decrease in the barrier height 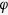. The lower barrier results in enhanced electron tunneling which leads to an enhanced radiative recombination with holes accumulated near the interface between SiO_2_ and p-GaN. This model as shown in the inset of Fig. 3(a) is confirmed by a strong correlation of barrier height reduction and emission intensity. The opposite phenomenon occurs when a negative gate voltage (V_G_) is applied. As shown in Figure S2, a negative gate voltage (V_G_) induces hole doping in the graphene that increases its work function and then leads to a larger barrier height 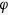 for electrons tunneling from graphene to p-GaN. This increase would cause a suppression of the EL intensity.

In the tunneling-based operating mechanism electrostatic gating introduces a varying density of state in the graphene without affecting the condition of the luminescent material. This advantage is demonstrated when analyzing the bias dependence of the emission spectrum. Commonly, p-GaN exhibits a blue-shift in emission for high bias that is caused by electric field-induced polarizations in the material^22^. In our device the wavelength is stable within 0.3 nm across the whole measured bias current range (Fig. 3(d)).

### The comparison of VtiLET between optics and electronics

**Figure 4:**
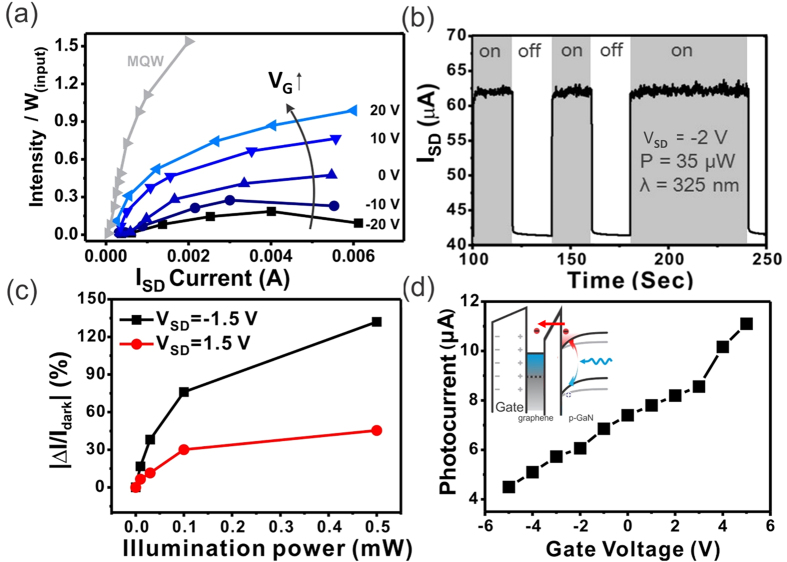
(**a**) The Normalized efficiency v.s. bias current under different application gate voltage compared with commercial MQW-LEDs. (**b**) Time resolved photoresponse under light (blue area) and dark condutions (*λ* = 325 nm). (**c**) Comparison of photoresponse under forward and reverse biases (V_SD_) between p-GaN and graphene layer. (**d**) Photocurrent under different gate voltage (V_G_) at fixed reverse bias of −1.5 V. (inset) Energy level diagram of the graphene/thin SiO_2_/p-GaN device with external positive gate voltage (V_G_) and reverse bias (V_SD_ < 0 V) under light excitation. With increasing positive gate voltage, the band bending of p-GaN near the interface will become more pronounced, which can enhance the separation of electrons and holes and increase the photocurrent.

The light emitting transistor structure employed here was found to improve the performance of light emitters. Commercially available LEDs based on multiple GaN/InGaN/GaN quantum wells exhibit a turn on voltage around 2.75 V, which necessitates high operating voltages and increases power consumption. In our device, applying a gate voltage decreases the turn on voltage to 1 V under positive gate voltage and increases it to 12 V for under negative gate voltage. Application of a gate thus increases the efficiency to ~200% compared to ungated conductions. This novel operating mechanism yields unoptimized emission efficiencies of ~50% of commercial multiple quantum well light emitting diodes (MQW-LEDs) (Fig. 4(a)), which could in the future be further enhanced through optimization of the graphene conductivity and the barrier thickness.

In addition to an enhanced emission, electrostatically controlled tunneling promises faster and more efficient transition between on- and off-states than conventional transistor operation mechanisms^7^,^38^. This behavior makes it ideally suited for high speed connection between optics and electronics. To take full advantage of this linkage, conversion between light and current should proceed in both directions and a receiver is needed. We here demonstrate that our graphene/oxide/semiconductor structure can not only emit but also detect light. This dual role can improve the integration density of optical communication devices and enable on-chip optical computing.

Figure 4(b) shows the emission current under time-varying illumination with a 325 nm laser. The underlying band bending mechanism results in a much faster response and higher sensitivity than normal GaN-based devices. The response time to achieve saturation is below 0.5 seconds regardless of the light excitation while traditional devices require minutes to reach saturation^39^,^40^. The photodetection mechanism was investigated by using different excitation sources. Figure S3 shows that the device has the largest photoresponse under 325 nm while 514 nm and 1064 nm show a much smaller response. In the case of the 1064 nm excitation, heating effects were identified as the origin of the response. We therefore conclude that excitation across the GaN band gap is responsible for the photoresponse. The small photoresponse of the 514 nm excitation source is attributed to contributions from defect transition^41^. Photogeneration of electron-hole-pairs is followed by separation of the carriers within the p-GaN region near the surface where the bands are bent. This mechanism was confirmed by comparing the sensitivity under forward (V_SD_ > 0 V) and reverse bias (V_SD_ < 0 V) (Fig. 4(c)). We observe that the reverse bias has a larger photocurrent signal ratio than forward bias due to the asymmetric band brought about by the opposing internal and external fields under forward bias.

Figure 4(d) demonstrates the ability to electrostatically tune the detectors sensitivity independent of applied bias. The mechanism of the electrostatically tunable sensitivity is illustrated in the inset of Fig. 4(d). When the gate voltage (V_G_) increases, the decrease of the work function of graphene will induce larger band bending of p-GaN. Due to the larger bending the excited electron and holes could be separated much easier and generate a higher photoresponse. The observed linearity in sensitivity with gate voltages improves the accuracy of the sensor compared to traditional photosensors which exhibit an exponential characteristic. The different dependences of photosensitivity on gate voltage (V_G_) and source-drain bias (V_SD_) could enable sensors that are adaptable to widely different illumination conditions (through control of V_SD_) while retaining ultrahigh sensitivity (by modifying V_G_). Compared with published reports of GaN based photodetectors, the phtoresponse shown here is more than a factor of 10 times higher^40^.

## Conclusion

In conclusion, we have utilized a heterojunction of 2D materials and ultrathin films in a novel and multifunctional device that serves as light emitter, light detector and transistor. The integration of these unique properties requires less complex fabrication and lend themselves to new applications. It was observed that carrier injection from graphene into a luminescent material created emission from well-defined states and its intensity could be controlled by a gate. The mechanism was found to be based on tunneling through a barrier that could be modified by changing the graphene work function. The application of a gate was shown to enhance the emission efficiency and the performance of the emitter and also enabled multifunctional devices. The presented gate controlled carrier injection mechanism enabled fine control of the device operation, such as tunable detection sensitivity. Finally, the universal applicability of the underlying quantum tunneling mechanism is compatible with a wide range of 2D and quasi-2D materials which enables tailoring of device properties for a bright future for optoelectronics.

## Methods

### Device fabrication and characterization measurements

Figure 1(a) shows the structure of the proposed tunneling injector structure. Mg doped GaN (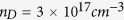 determined by Hall measurement) on sapphire acts as the luminescent material and the substrate which was grown by metal organic chemical vapor deposition. Before fabrication it was cleaned following established procedures^10^.

A 10 nm thin SiO_2_ film was deposited on the p-GaN surface as a tunneling barrier via radio frequency (RF) sputtering. Finally, graphene grown by chemical vapor deposition on copper foil was transferred onto the SiO_2_ with the aid of polymethylmethacrylate (PMMA) following previous reports^42^,^43^. The quality and thickness of the transferred graphene were verified by Raman spectroscopy (Jobin Yvon T64000) (Fig. 1(b))^44^,^45^. The Raman signal of graphene on p-GaN shows additional strong peaks at 573 cm^−1^ which is the characteristic phonon frequency of the GaN and at 737 cm^−1^ which was previously found to originate from Mg^+2^ dopants^46^. Finally, the broad background is due to the photoluminescence (PL) effect from p-GaN. A solid gate dielectric layer was deposited on top of the graphene. To avoid damage to the graphene, we first used electron beam evaporation to obtain a 30 nm thin SiO_2_ film that protects the graphene and then sputtered 300 nm thick SiO_2_ film by RF sputtering. To keep the quality of this stacking structure without the damage from one more water transfer of graphene, dry transfer process of graphene onto the thick SiO_2_ as a transparent top-gate electrode which was followed with previous report^47^. After that, Au/Ni for probe contacts which were subsequently deposited by electron beam evaporation at a pressure of ~6 × 10^−7^ Torr and at a constant sample rotation rate. To improve the deposited electrode’s contact resistance, annealing was carried out in a furnace at 400 °C for 20 minutes.

## Additional Information

**How to cite this article**: Tan, W.-C. *et al*. Tunneling-injection in vertical quasi-2D heterojunctions enabled efficient and adjustable optoelectronic conversion. *Sci. Rep.* **6**, 31475; doi: 10.1038/srep31475 (2016).

## Supplementary Material

Supplementary Information
