# Supplementary material for: Tunneling-injection in vertical quasi-2D heterojunctions enabled efficient and adjustable optoelectronic conversion
Source: Sci Rep. 2016 Aug 10;6:31475. doi: 10.1038/srep31475 (PMC4979038; doi:10.1038/srep31475)
Supplement: Supplementary Information [file srep31475-s1.pdf]

Supplementary Information

Tunneling-injection in vertical quasi-2D heterojunctions enabled efficient and adjustable optoelectronic conversion

Wei-Chun Tan,[1] Chia-Wei Chiang,[1] Mario Hofmann[2]* and Yang-Fang Chen[1]*
***Corresponding authors: Yang-Fang Chen & Mario Hofmann**

1 Department of Physics, National Taiwan University, Taipei, Taiwan 10617
E-mail: yfchen@phys.ntu.edu.tw
2 Department of Material Science and Engineering, National Cheng Kung University, Tainan, Taiwan 70101
E-mail: mario@mail.ncku.edu.tw
Keywords: graphene、light emission、tunneling、transistor、photodetector

S. 1

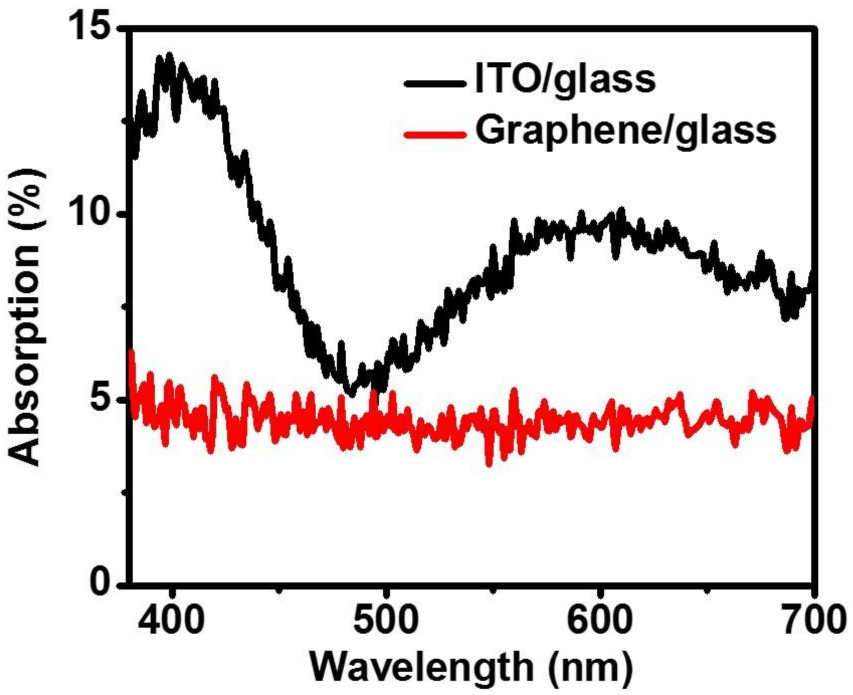

Figure S. 1 Comparison of absorption spectra from ITO and SLG graphene on glass substrate.

S. 2

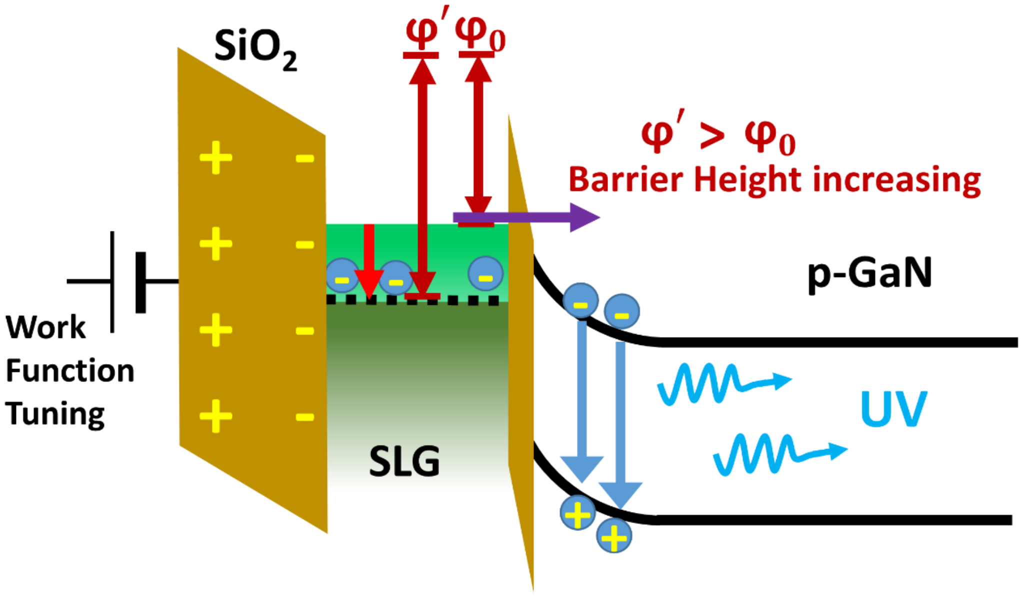

Figure S. 2 Energy level diagram of the graphene/thin SiO_2/p-GaN device under negative external gating (V_G) and forward bias (V_{SD} > 0 V).

S. 3

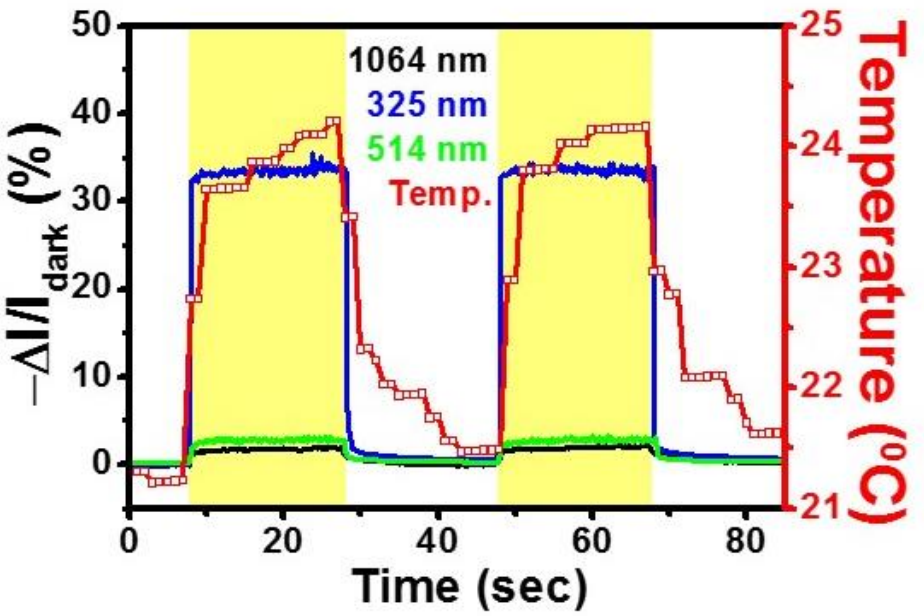

Figure S. 3 The photoresponse during light-on (yellow) and light-off under the consistent power illumination of three different laser with the corresponding temperature evolution.

S. 4

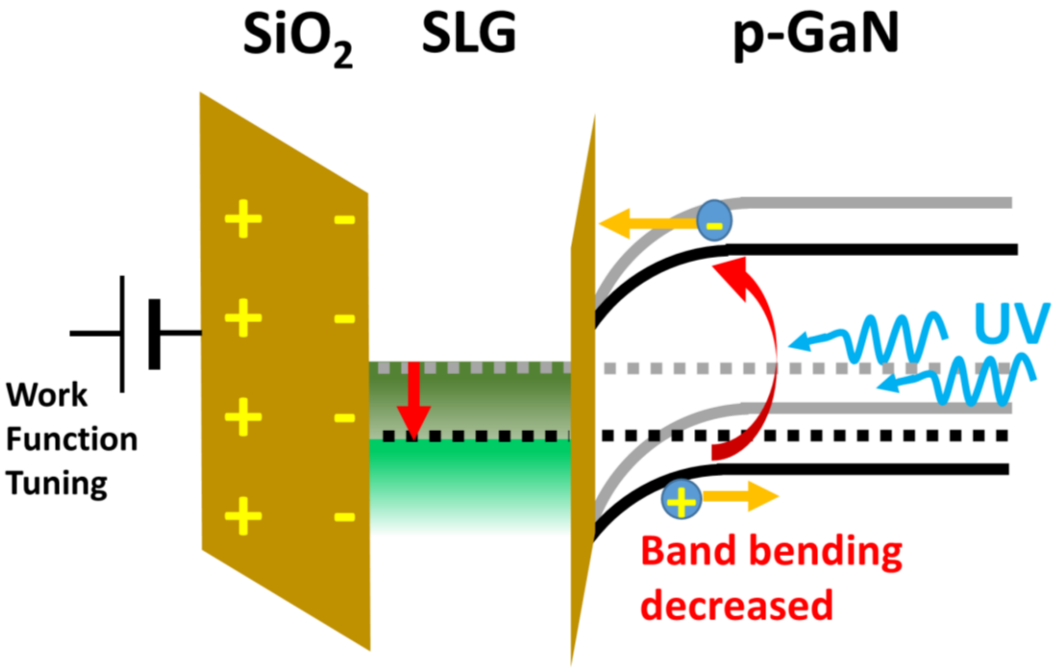

Figure S. 4 Energy level diagram of the graphene/thin SiO_2/p-GaN device with external negative gate voltage (V_G) and reverse bias (V_{SD} < 0 V) under light excitation: tuning work function of graphene inhibits the photoresponse.
